# New Remedies

**Published:** 1887-04

**Authors:** A. W. Harlan

**Affiliations:** Chicago


### New Remedies.

BY A. W. HARLAN, M.D., D.D.S., CHICAGO.

[Read at the Forty-third Annual Meeting of the Mississippi Valley Dental Association.]

There have been a number of drugs brought to our notice during the past few years, some of which have already been adopted in the daily practice of many dentists. Very often dentists, like doctors, read of the supposed wonders that a new drug will accomplish, and pass it by for the time being, expecting to use it when occasion offers. This may not be for a long time, if the drug is not easily attainable, is high-priced, or its properties not well understood. If we look back for only a short period, it will be noticed that as long as cocaine was scarce, there was a steady demand for it, but now I fancy there are many dentists who, after their curiosity was satisfied, have ceased using it. .It does not follow that something better has supplanted it, they were disappointed in its use because it would not act uniformly in all cases. For a variety of reasons, either the drug was impure, lacked potency, or it was improperly exhibited; at any rate, it could not obtund sensibility in dentine, and there was the place where it was most needed. Cocaine then and now is of value, but it has not a wide range of usefulness in the daily practice of dentistry. The novelty has worn off, and it is finding its legitimate place as a local anæsthetic for the soft tissues.

The newer remedies are iodol, aseptol, eulyptol, and trichlorphenol. Of the first, if it was intended to supplant iodoform, it has one virtue in not being nauseous to smell. I have used it, combined with pure terepen, to paint the edges of ragged gums, as a dressing after the removal of necrosed bone, and have packed it in pockets around teeth to check the production of pus. In all such cases it may be substituted for iodoform. In combination with oil of wintergreen, packed into foul smelling roots, it acts with certainty, destroying the odor and rendering them aseptic in from three to five days. It is useful in teeth with exposed pulps, causing no pain, but very quickly subduing it.

It may be packed into indurated folds of the gums or mucous membrane, and after a short period such tissues become less rigid, and regain their normal sensibility and mobility. Aseptol or sogolic acid is an oily liquid with an acid reaction, soluble in water and other menstrum, and is a powerful germicide and disinfectant. I have made some experiments with it in the dressing of foul ulcers, and also in sinuses leading from necrosed bone. When it has been applied to inflamed gums and injected between them and the roots of teeth, the redness and soreness have quickly disappeared. It is a stimulant locally when diluted to one in ten, or even one in forty. It may be injected into and through roots of teeth with fistulous opening, always largely diluted. It will cause pain if applied in full strength to an exposed pulp, but by adding an essential oil, such as cloves, — or gaultheria, it quickly becomes an anodyne and stimulant, and a very feeble coagulator of albumen. As a spray in a two per cent. solution for the nose or throat, it is admirable, as well as for irrigating the antrum. Eulyptol is composed of six parts salicylic acid, two parts crystals carbolic acid, and one part eucalyptol. This makes a consistent paste, which will stick to the neck of a tooth or the gum (if previously dried) for a longer time than any other medicant with which I am acquainted. I have used it in treatment of sensitive necks of teeth with success. Its only rival is the bleo-resin of kava-kava. It is a powerful disinfectant, but not so potent as aseptol. It has been used experimentally for obtunding dentine, but, as yet, it does not offer to rank with cocaine, ether, or the fluid extract of cannibas indica.

It may be smeared into a pulp chamber and covered with cotton soaked in a gutta-percha solution and allowed to remain for a few days before using a broach in a canal to remove putrid matter with success. It is a deodorizer and preservative, and if it has no further good quality than the one of adhering to the neck of a tooth and removing the sensitiveness at that point, it is a valuable addition to our list. Trichlor-phenol contains three atoms of chlorine in place of hydrogen in carbolic acid. It is insoluble in water, but freely so in alcohol, ether, essential oils, glycerine, etc. I prefer solvents in the order named, glycerine, essential oils, alocohol, and ether. It is stated to be twenty-five times more powerful than carbolic acid, hence weak solutions are to be prepared. It checks the production of pus, is a stimulant and non-irritant in solutions of less than twenty per cent., and may be used whenever iodoform is indicated as antiseptic dressing and a certain disinfectant. Its weak point is non-solubility in water. It forms soluble salts with bases, and on this account may prove of value in the treatment of phagedena.

Some one may ask why we should try new and in some cases untried remedies when there are many old and prompt acting agents for use in the treatment of all known diseases. My answer would be, efficiency, potency, non-poisonous properties, which are all sought in the order named, and when a new drug possesses these, it is our duty to substitute it for the old, proving our interest in progress, and a determination to base practice on fixed principles of scientific advancement.

